# Ten Years of Change in Bariatric/Metabolic Surgery in the Asia–Pacific Region with COVID-19 Pandemic: IFSO-APC National Reports 2021

**DOI:** 10.1007/s11695-022-06182-x

**Published:** 2022-06-30

**Authors:** Masayuki Ohta, Soo Min Ahn, Yosuke Seki, Wah Yang, Simon Kin-Hung Wong, Suthep Udomsawaengsup, Jeffrey M. Hamdorf, Manish Khaitan, Nik Ritza Kosai, Weu Wang, June Lee, Reno Rudiman, Thejana Wijeratne, Edward Oliveros, Cunchuan Wang, Kazunori Kasama

**Affiliations:** 1 Research Center for GLOBAL and LOCAL Infectious Diseases, Oita University, 1-1 Idaigaoka, Hasama-machi, Yufu, Oita, 879-5593 Japan; 2 Departments of Gastroenterological and Pediatric Surgery, Faculty of Medicine, Oita University, Yufu, Japan; 3 Department of Pediatric Surgery, Severance Obesity Surgery Center, Gangnam Severance Hospital, Yonsei University College of Medicine, Seoul, Korea; 4 Weight Loss and Metabolic Surgery Center, Yotsuya Medical Cube, Tokyo, Japan; 5 Department of Metabolic and Bariatric Surgery, the First Affiliated Hospital of Jinan University, Guangzhou, China; 6 Division of Upper Gastrointestinal & Metabolic Surgery, Department of Surgery, Faculty of Medicine, The Chinese University of Hong Kong, Hong Kong, China; 7 Department of Surgery, Faculty of Medicine, Chulalongkorn University, Bangkok, Thailand; 8 Clinical Training and Evaluation Centre, Medical School, The University of Western Australia, Crawley, Australia; 9 Nobesity Bariatric Center, KD Hospital, Ahmedabad, India; 10 Minimally Invasive Upper Gastrointestinal and Bariatric Surgery Unit, Department of Surgery, Universiti Kebangsaan Malaysia Medical Centre, Kuala Lumpur, Malaysia; 11 Department of Surgery, School of Medicine, College of Medicine, Taipei Medical University, Taipei, Taiwan; 12 Division of General Surgery, Department of Surgery, Taipei Medical University Hospital, Taipei, Taiwan; 13 Department of General Surgery, Changi General Hospital, Singapore, Singapore; 14 Division of Digestive Surgery, Department of General Surgery, School of Medicine, Universitas Padjadjaran, Hasan Sadikin General Hospital, Bandung, Indonesia; 15 Department of Surgery, Faculty of Medical Sciences, University of Sri Jayawardenepura, Nugegoda, Sri Lanka; 16 Institute of Surgery, St. Luke’s Medical Center, Manila, Philippines

**Keywords:** Bariatric/metabolic surgery, Asia–Pacific region, Indication, Insurance coverage, COVID-19

## Abstract

**Background:**

On November 25, 2021, the IFSO-Asia–Pacific Chapter (IFSO-APC) Virtual Meeting 2021 was held online, and the representatives from the Asia–Pacific region presented 10 years of change in bariatric/metabolic surgery and the influence of COVID-19 in the special session of “IFSO-APC National Reports 2010–2020”. We herein report the summarized data.

**Methods:**

National bariatric/metabolic surgery data, which included the data of 2010 and 2020, were collected from the representatives using a questionnaire that consisted of 10 general questions. At the congress, the data were calculated and summarized.

**Results:**

Thirteen of the 14 national societies responded to the survey. From 2010 to recent years, the populations of individuals with obesity (BMI ≥ 30 kg/m^2^) and individuals with diabetes both significantly increased. Eight countries and regions expanded the lower limit of criteria for bariatric surgery by 2–5 kg/m^2^ (BMI), and 5 countries newly established criteria for metabolic surgery in the last ten years. Sixty-nine percent of the countries currently run public health insurance systems, which doubled from 2010. The number of bariatric surgeons and institutions increased more than threefold from 2010. In 2010, 2019, and 2020, surgeons in IFSO-APC societies performed 18,280, 66,010, and 49,553 bariatric/metabolic surgeries, respectively. Due to the COVID pandemic, restriction policies significantly reduced access to surgery in South and Southeast Asian countries. The biggest changes included increased numbers of bariatric surgeons and institutions, operation numbers, public insurance coverage, raising awareness, and national registry systems.

**Conclusion:**

For the last 10 years, bariatric/metabolic surgery has rapidly grown in the Asia–Pacific region.

## Introduction

The International Federation for the Surgery of Obesity and Metabolic Disorders (IFSO) is a federation of societies of bariatric and metabolic surgery in each country and region. The Asia–Pacific Chapter (APC) was founded in 2008, and 4 national societies joined as members. The first congress of the IFSO-APC was held in 2009 in Cairns, Australia, and the second was held in 2011 in Rusutsu, Japan. IFSO-APC 2011 included a consensus meeting for indications for bariatric/metabolic surgery, and a national reports session was first attempted in the IFSO-APC [1]. The consensus statement showed that the indication for bariatric surgery in Asian candidates is a body mass index (BMI) ≥ 35 kg/m^2^, and that the indications for metabolic surgery are BMI ≥ 30 kg/m^2^ with type 2 diabetes mellitus (T2DM) or metabolic syndrome [2]. National societies in Middle Eastern countries subsequently joined the IFSO-APC. In 2017, with the establishment of the IFSO-Middle East North Africa Chapter, these societies withdrew from IFSO-APC.

The IFSO worldwide survey has been repeatedly performed since 1997 [3]. Only 2 to 5 national societies in the current IFSO-APC responded to the IFSO surveys until 2011 [3–6]; however, 8 national societies submitted completed forms in the 2013 IFSO survey [7]. The survey showed that 28,773 patients underwent bariatric/metabolic surgery in Asia–Pacific countries and regions. According to the IFSO worldwide survey in 2018, 70,573 patients received bariatric/metabolic surgery in 11 countries in the IFSO-APC, including Iran [8]; this number rapidly increased by 2.5-fold over 5 years. In addition, the 2018 IFSO survey first showed the presence or absence of national guidelines, preoperative routine gastroscopy, and reimbursement.

On November 25, 2021, the IFSO-APC Virtual Meeting 2021 was held online, and a special session, “IFSO-APC National Reports 2010–2020,” presented 10 years of change in bariatric/metabolic surgery and the influence of COVID-19 in the IFSO-APC countries. We herein report the summarized data as an official task force in the IFSO-APC.

## Methods

The questionnaire and template of the presentation slide were prepared and sent, three times, to the representatives of 14 national societies in the IFSO-APC by e-mail before the congress. The 14 countries and regions were as follows: Japan, Korea, China, Taiwan, Hong Kong, Philippines, Malaysia, Singapore, Indonesia, Thailand, India, Sri Lanka, Australia, and Iran. The questionnaire included the data of 2010 and 2020, and consisted of 10 general questions:

1. What was the prevalence of obesity (BMI ≥ 30 kg/m^2^) and T2DM in your country in 2010 and in recent years?
2. What were the indications for bariatric and metabolic surgeries in your country in 2010 and 2020?
3. Were bariatric and/or metabolic surgeries covered by the public health insurance in your country in 2010 and 2020?
4. How many bariatric surgeons and institutes worked in your country in 2010 and 2020?
5. How many patients received bariatric/metabolic surgery in your country in 2010, 2019 and 2020?
6. How many patients received each bariatric/metabolic surgery procedure in your country in 2010, 2019 and 2020?
7. Which were the data sources in your country in 2010, 2019 and 2020; (i) national registry of the society for bariatric/metabolic surgery or medical funding system, (ii) national survey conducted by the society for bariatric/metabolic surgery, or (iii) personal communication?
8. What were the biggest changes in bariatric/metabolic surgery in your country between 2010 and 2020?
9. What are current problems and future perspective on bariatric/metabolic surgery in your country?
10. How did COVID-19 influence bariatric/metabolic surgery in your country?

Therefore, this survey covered two aspects: (1) change in epidemiological and statistical data and (2) the influence of COVID 19. All the slides presented in IFSO-APC National Reports 2011 [1] were sent to the representatives from Japan, Korea, Taiwan, Hong Kong, Philippines, Malaysia, Singapore, Indonesia, Thailand, India, and Australia as references for the 2010 data before the 2021 congress. With the exception of Taiwan and Hong Kong, the population of individuals with obesity in each country and region was obtained from the World Health Organization (WHO) database [9]. For Taiwan and Hong Kong, these populations were obtained from other documents and databases [10–12]. The population of individuals with T2DM was obtained from the International Diabetes Federation (IDF) Diabetes Atlas [13, 14]. The population of individuals with obesity was reported in the WHO database until 2016; the most recent year was 2016. The population of individuals with T2DM was reported in the IDF Diabetes Atlas until 2021; the most recent year was 2021.

Each representative agreed on describing the manuscript using the slides. At the congress, the data were calculated and summarized. After the congress, the data were updated for further analysis by repeated e-mails, which were exchanged with the representatives. The order of the countries in the tables and figures was arranged as follows: East Asia, Southeast Asia, South Asia, and Oceania.

The population of individuals with obesity and T2DM in IFSO-APC countries and regions was analyzed and expressed as the average (range). Data on the average prevalence of obesity and T2DM and the numbers of bariatric surgeons and institutions in each country and region were evaluated using the Wilcoxon signed-rank test, and data on the number of bariatric/metabolic surgeries performed in 2010, 2019, and 2020 in each country were evaluated using Friedman’s test with Bonferroni correction for multiple comparisons. *P* values of < 0.05 were considered to be statistically significant. All statistical analyses were performed using the Statistical Package for the Social Science (SPSS) II software program (version 26, SPSS, Inc., Chicago, IL, USA).

## Results

Thirteen representatives from each country and region reported their national data at the congress. Iran did not present their national data. Data were supplemented afterward and analyzed.

### Change in the Prevalence of Obesity and T2DM in the General Population

**Fig. 1:**
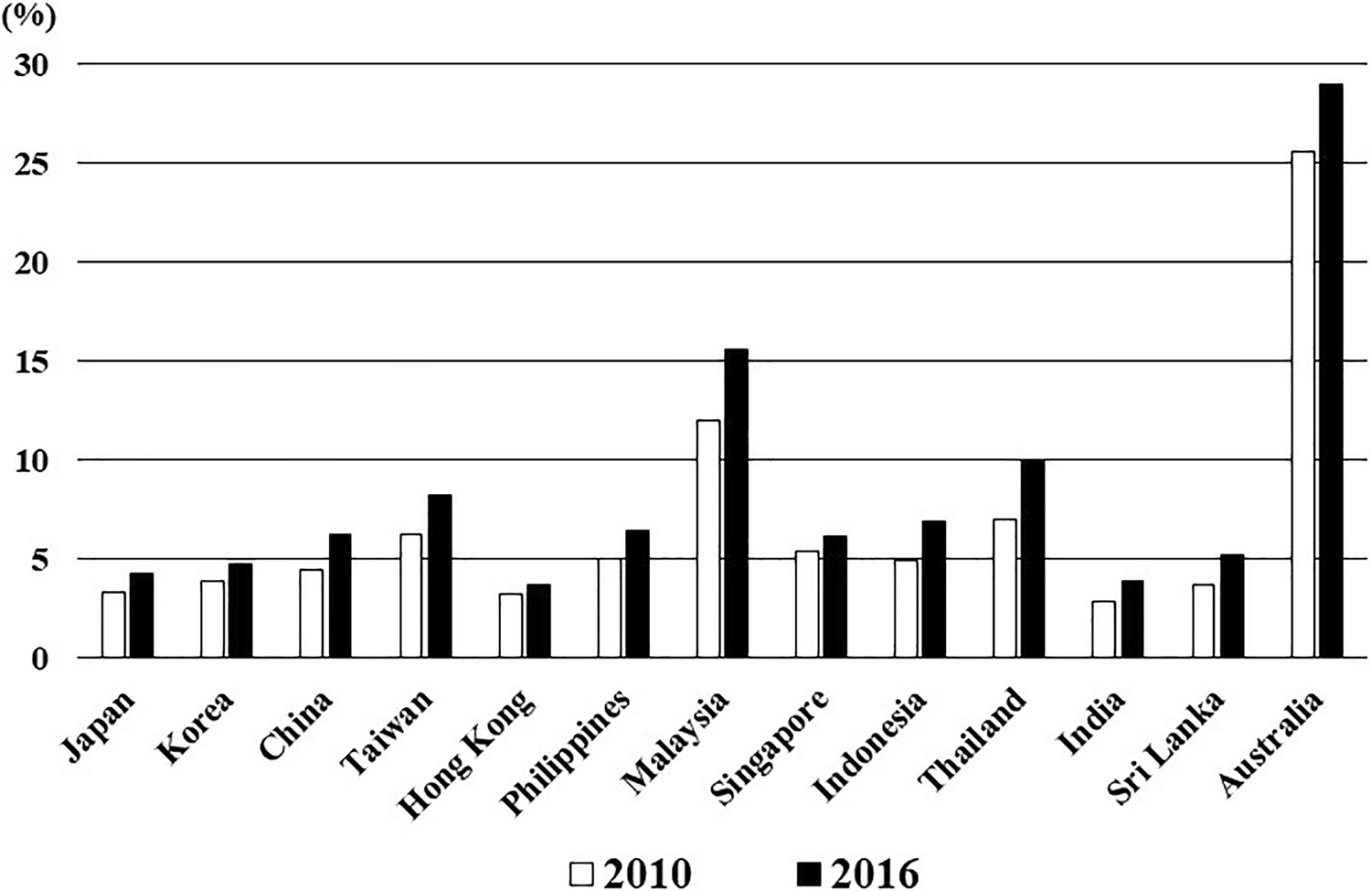
Change in the prevalence of individuals with obesity (BMI ≥ 30 kg/m.^2^)

**Fig. 2:**
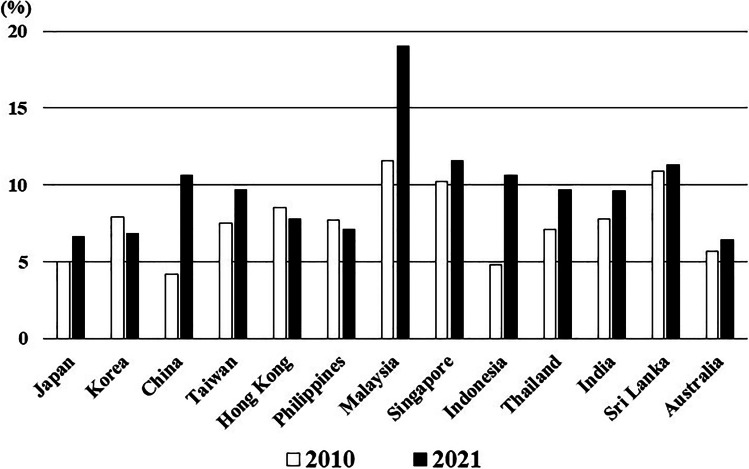
Change in the prevalence of individuals with type 2 diabetes mellitus

The average prevalence of obesity (BMI ≥ 30 kg/m^2^) in the general population in 2010 and 2016 was 6.7% (2.8–25.6%) and 8.5% (3.7–29.0%), respectively, and the population of individuals with obesity significantly increased in all of the countries (*p* = 0.001, Fig. 1). The average prevalence of T2DM in the general population in 2010 and 2021 was 7.6% (4.2–11.6%) and 9.8% (6.4–19.0%), respectively, and the population of individuals with T2DM significantly increased in 10 of the 13 countries (76.9%) (*p* = 0.014, Fig. 2).

### Changes in the Indications for Bariatric and Metabolic Surgeries, and Public Health Insurance Coverage of Bariatric and Metabolic Surgeries

Changes in the indications for bariatric and metabolic surgeries are shown in Table 1. In 8 of the 13 countries (61.5%), the indications for bariatric surgery were expanded from 2010 to 2020. Indications for metabolic surgery were newly established in 5 of the 13 countries (38.5%) until 2020 and were expanded in 4 countries (30.8%). At present, in many IFO-APC countries and regions, the indication for bariatric surgery is BMI ≥ 35–37.5 kg/m^2^, and the indications for metabolic surgery are BMI ≥ 27.5–32 kg/m^2^ with T2DM or other 2 obesity-related diseases.

**Table 1:** Changes in the indications for bariatric and metabolic surgeries in 2010 and 2020

Countries or regions	Indication of bariatric surgery (BMI)		Indication of metabolic surgery (BMI)	
	2010	2020	2010	2020
Japan	≥ 35	≥ 35	≥ 32 with a comorbidity	≥ 32 with T2DM or other 2 comorbidities
Korea	≥ 40	≥ 35	≥ 35 with a comorbidity	≥ 30 with a comorbidity or ≥ 27.5 with T2DM
China	≥ 40 or ≥ 35 with comorbidities	≥ 32.5	None	≥ 27.5 with T2DM
Taiwan	≥ 40 or ≥ 35 with comorbidities	≥ 37.5 or ≥ 32.5 with T2DM or other 2 comorbidities	None	≥ 27.5 with poorly controlled T2DM
Hong Kong	≥ 37.5 or ≥ 32.5 with comorbidities	≥ 35 or ≥ 30 with comorbidities	None	≥ 27.5 with poorly controlled T2DM
Philippines	≥ 35 with comorbidities	≥ 35	None	≥ 32 with T2DM or other 2 comorbidities
Malaysia	≥ 40	≥ 37.5	≥ 35 with T2DM or other 2 comorbidities	≥ 32 with T2DM or other 2 comorbidities
Singapore	≥ 37.5	≥ 37.5	≥ 32.5 with a comorbidity	≥ 32.5 with a comorbidity
Indonesia	> 37	≥ 35	> 32 with comorbidities	> 30 with a comorbidity
Thailand	≥ 37.5	≥ 37.5	≥ 32.5 with a comorbidity	≥ 32.5 with T2DM or other 2 comorbidities
India	≥ 40	≥ 35	≥ 35 with comorbidities	≥ 30 with T2DM or other 2 comorbidities
Sri Lanka	≥ 40	≥ 35	None	None
Australia	≥ 40 or ≥ 35 with comorbidities	≥ 40 or ≥ 35 with comorbidities	None	≥ 30 with T2DM or ≥ 32.5 with Asian

*T2DM*, type 2 diabetes mellitus; *BMI*, body mass index (kg/m^2^)

The changes in public health insurance coverage of bariatric and metabolic surgeries are also shown in Table 2. Regarding bariatric surgery, 4 of the 13 countries (30.8%) already had insurance coverage in 2010, and 5 countries (38.5%) gained coverage until 2020. The remaining 4 countries do not have coverage at the time of writing. Regarding metabolic surgery, 3 of the 13 countries (23.0%) already had insurance coverage in 2010, 5 (38.5%) gained coverage until 2020, and 5 (38.5%) do not have coverage at the time of writing. At present, in 9 of the 13 IFSO-APC countries (69.2%), either bariatric or metabolic surgery is covered by public health insurance, while 4 countries (30.8%) do not have coverage of bariatric/metabolic surgery at the time of writing. In other words, the percentage of countries in which bariatric/metabolic surgery is covered by public health insurance doubled from 2010.

**Table 2:** Changes in public health insurance coverage of bariatric and metabolic surgeries in 2010 and 2020

Countries or regions	Bariatric surgery		Metabolic surgery	
	2010	2020	2010	2020
Japan	Not covered	Covered (SG), partially covered (SG-DJB)	Not covered	SG for 32.5 ≤ BMI < 35 with T2DM limitedly covered
Korea	Not covered	Covered	Not covered	BMI ≥ 30 with a comorbidity covered, 27.5 ≤ BMI < 30 with T2DM partially covered
China	Not covered	Not covered	Not covered	Not covered (covered in some provinces)
Taiwan	Covered (VBG)	Covered (SG, RYGB)	Not covered	Not covered
Hong Kong	Not covered	Covered (support service in public hospitals)	Not covered	Covered (support service in public hospitals)
Philippines	Not covered	Not covered	Not covered	Not covered
Malaysia	Not covered	Not covered (reimbursement system)	Not covered	Not covered (reimbursement system)
Singapore	Covered (government-mandated medical savings plan)	Covered (government-mandated medical savings plan)	Covered (government-mandated medical savings plan)	Covered (government-mandated medical savings plan)
Indonesia	Not covered	Not covered	Not covered	Not covered
Thailand	Not covered	Covered	Not covered	Covered
India	Not covered	Covered (BMI ≥ 40)	Not covered	Covered (BMI ≥ 35 with a severe comorbidity)
Sri Lanka	Covered	Covered	Covered	Covered
Australia	Covered	Covered	Covered	Covered

*SG*, sleeve gastrectomy; *SG-DJB*, sleeve gastrectomy with duodenojejunal bypass; *BMI*, body mass index (kg/m^2^); *T2DM*, type 2 diabetes mellitus; *VGB*, vertical banded gastroplasty; *RYGB*, Roux-en-Y gastric bypass

### Changes in the Numbers of Bariatric Surgeons and Institutions, and the Numbers of Bariatric/Metabolic Surgery in 2010, 2019, and 2020

Changes in the numbers of bariatric surgeons and institutions in 2010 and 2020 are shown in Table 3. In the 13 IFSO-APC countries and regions, there were 466 bariatric surgeons and 296 bariatric institutions in 2010, and 1875 bariatric surgeons and 932 institutions in 2020; this amounted to a significant increase of a 4.0-fold increase in the number of bariatric surgeons and a 3.1-fold increase in the number of institutions (both *p* = 0.001). The number of bariatric surgeons and institutions increased in all of the countries. In China, in particular, there was a ≥ tenfold increase in the number of bariatric surgeons and institutions.

**Table 3:** Changes in the numbers of bariatric surgeons and institutions in 2010 and 2020

Countries or regions	No. of bariatric surgeons and institutions			
	Bariatric surgeons		Bariatric institutions	
	2010	2020	2010	2020
Japan	13	70	11	65
Korea	9	87	9	66
China	50	650	40	400
Taiwan	75	123	33	54
Hong Kong	10	28	4	10
Philippines	15	30	5	20
Malaysia	8	38	5	33
Singapore	15	37	5	12
Indonesia	4	17	6	10
Thailand	15	56	12	35
India	100	456	15	40
Sri Lanka	2	6	1	3
Australia	150	277	150	184
Total	466	1875	296	932

**Fig. 3:**
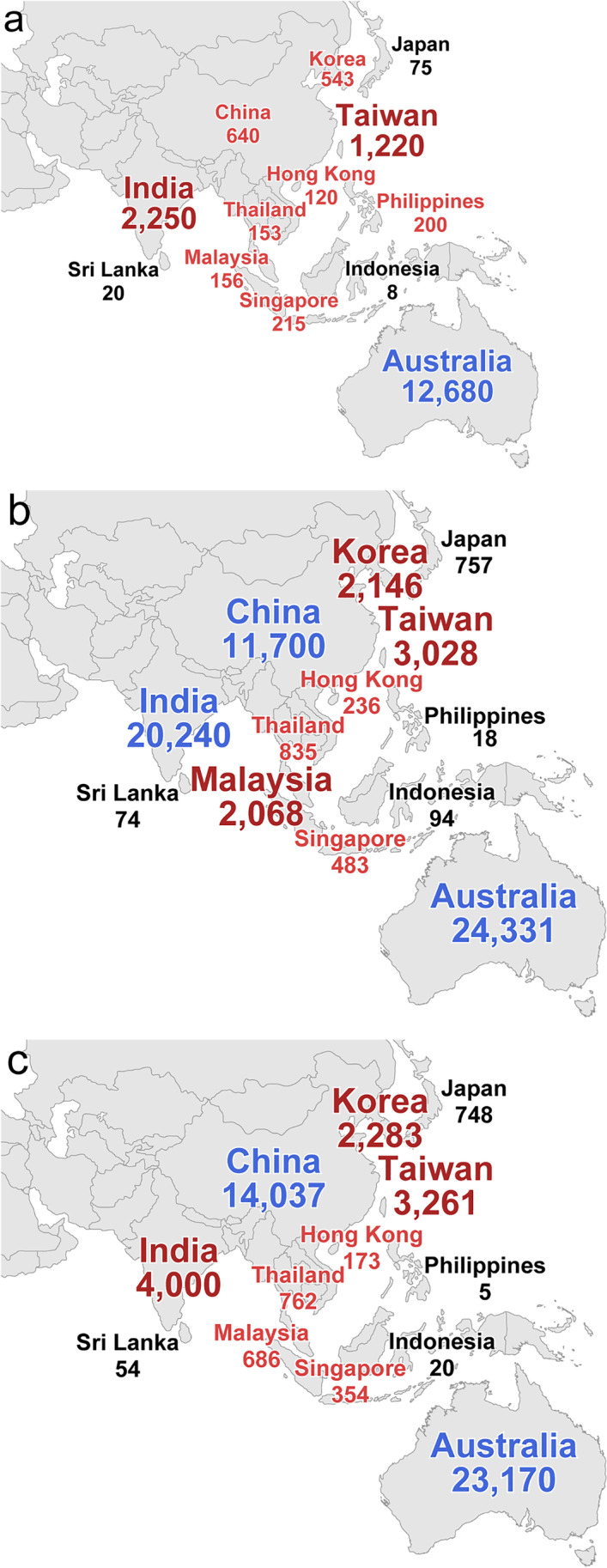
Changes in the numbers of bariatric/metabolic surgery in 2010 (**a**), 2019 (**b**), and 2020 (**c**)

Changes in the numbers of bariatric/metabolic surgery in 2010, 2019, and 2020 are shown in Fig. 3. In the IFSO-APC countries and regions, a total of 18,280 patients underwent bariatric/metabolic surgery in 2010; this number significantly increased to 66,010 in 2019 (*p* < 0.001) and 49,553 in 2020 (*p* = 0.032), which amounted to a 3.6-fold increase and a 2.7-fold increase, respectively. However, there was no significant difference in the total number between 2019 and 2020 (*p* = 0.509). Notably, in Japan, China, and Malaysia, the number of cases in 2019 increased by more than tenfold in comparison to 2010. In Southeast and South Asian countries, there was a definite decrease in the number of cases in 2020. In contrast, in other countries, the number was increased or slightly decreased. In other words, it is considered that COVID-19 influenced bariatric/metabolic surgery in Southeast and South Asian countries more than in the other countries.

**Fig. 4:**
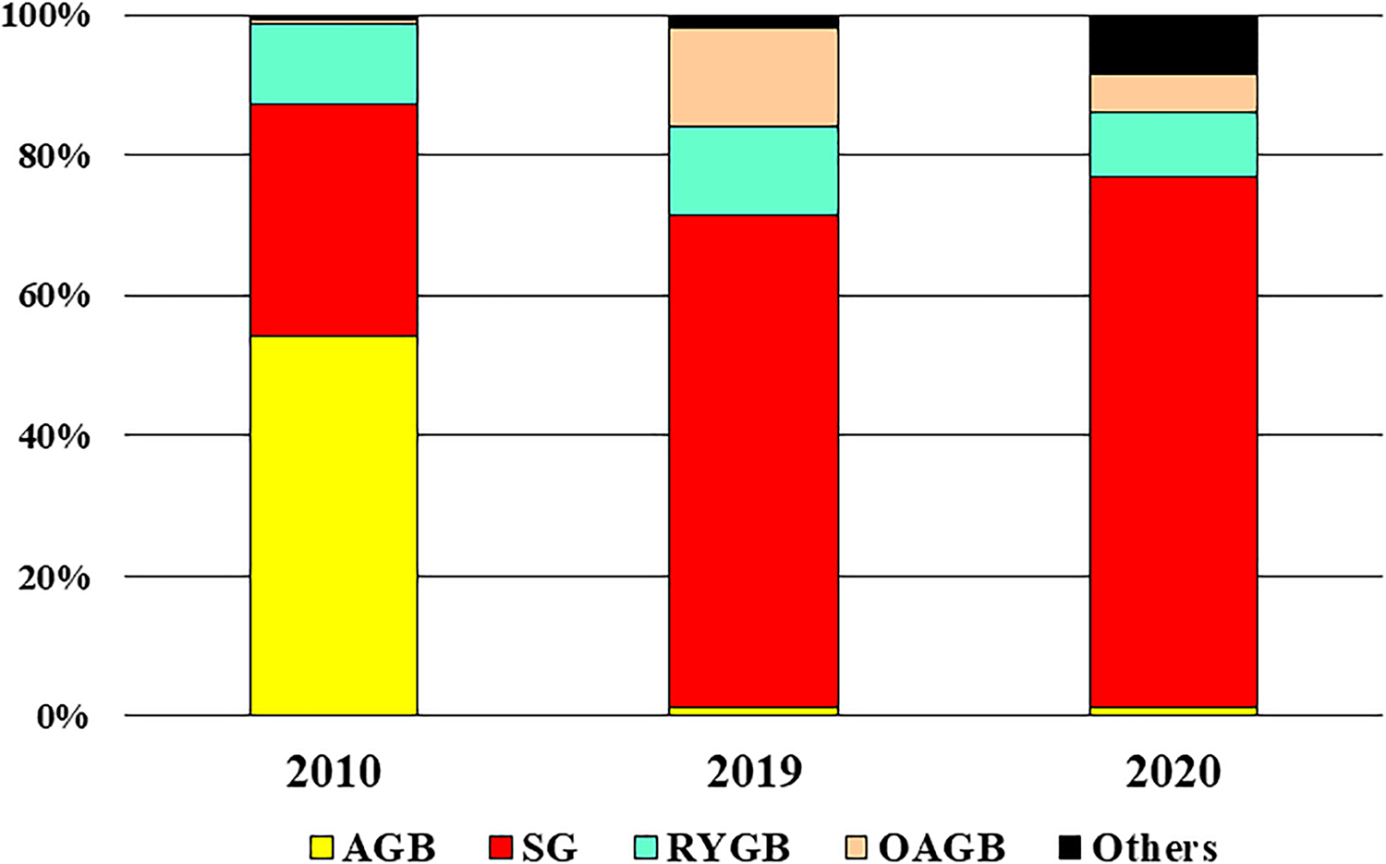
Percentages of each bariatric/metabolic procedure in 2010, 2019, and 2020. The 2010 data did not include the data of Indonesia and Sri Lanka, and the 2020 data did not include the data of India. AGB, adjustable gastric banding; SG, sleeve gastrectomy; RYGB, Roux-en-Y gastric bypass; OAGB, one anastomosis gastric bypass

The details of the procedures in Indonesia and Sri Lanka in 2010, and in India in 2020 were unknown. The percentages of each bariatric/metabolic procedure in the 13 IFSO-APC countries and regions are shown in Fig. 4. In 2010, adjustable gastric banding (AGB) accounted for 54.2% of the procedures, sleeve gastrectomy (SG) accounted for 33.0% and Roux-en-Y gastric bypass (RYGB) accounted for 11.7%. However, in 2019, SG accounted for 67.0% of the procedures, one anastomosis gastric bypass (OAGB) accounted for 13.5% and RYGB accounted for 12.1%. In the 2020 data—not including the Indian data—SG accounted for 75.7% of the procedures, RYGB accounted for 9.1% and OAGB accounted for 5.5%. These data clearly demonstrated that AGB rapidly decreased and that SG and OAGB increased for 10 years.

The data sources in 2010 were the national registry in one country (7.7%), national surveys in 8 countries (61.5%), and personal communication in 4 countries (30.8%). However, in 2019, the data from 4 countries (30.8%) were based on the national registries, the data from 6 countries (46.2%) were based on national surveys, and the data from 3 countries (23.0%) were based on personal communication. In 2020, the data sources in 3 countries (23.0%) were the national registries, the data sources in 7 countries (53.8%) were national surveys, and the data sources in 3 countries (23.0%) were personal communication. The percentage of the number of bariatric/metabolic surgeries based on personal communication to the total number in the Asia–Pacific region was only 1.9% in 2010, 0.3% in 2019, and 0.2% in 2020, respectively.

### The Biggest Changes in Bariatric/Metabolic Surgery in the 10-Year Period from 2010 to 2020

Seven of the 13 countries (53.8%) responded that the biggest changes in the last 10 years were increased numbers of bariatric surgeons and institutions. Seven countries (53.8%) also responded that there was an increased number of bariatric/metabolic cases. In 6 countries (46.2%), public insurance coverage of bariatric/metabolic surgery was established or expanded in the last 10 years. Raising awareness about bariatric/metabolic surgery was cited in 3 countries (23.1%), and a national registry system was established in 3 countries (23.1%). The other major changes included the establishment of societies, guidelines, journals, and a certified bariatric nurse board. A certified bariatric nurse board was cited in Taiwan.

### Current Problems and Future Perspective on Bariatric/Metabolic Surgery

In 10 of the 13 countries (76.9%) including 6 countries that already had public insurance coverage of bariatric/metabolic surgery, insurance coverage and medical funding systems were cited as a problem. Lack of awareness and comprehension of bariatric/metabolic surgery among physicians and the general public was cited as a problem in the 5 countries (38.4%). A small number of cases was cited as a problem in 5 countries (38.4%). Other problems included training systems, medical expenses, a lack of institutions and human resources, induction of new techniques, database utilization, the spread of metabolic surgery, and the concept of obesity.

### Influence of COVID-19 on Bariatric/Metabolic Surgery

According to the changes in the data in 2019 and 2020, 8 of 13 countries (61.5%) responded that bariatric/metabolic surgery was temporarily or entirely restricted, and as a result, the total number of cases decreased. The remaining 5 countries (38.5%) responded that the total number of cases was almost unchanged or increased. Therefore, between 2019 and 2020, operative cases decreased by 24.9% due to the impact of COVID-19 in South and Southeast Asia.

## Discussion

This survey clearly demonstrated that in the Asia–Pacific region, the number of bariatric surgeons and institutions increased by more than threefold in 10 years. The number of bariatric/metabolic surgeries also increased by more than 2.5-fold. The biggest changes in this period were the establishment of public health insurance coverage, raising awareness about bariatric/metabolic surgery, and the national registry systems. As sporadically revealed in the reports from several individual countries [15–18], bariatric/metabolic surgery has been rapidly developing in Asia–Pacific countries and regions for the last 10 years. This survey also clearly demonstrated that contrary to the rapid decrease in AGB, the numbers of SG and OAGB increased in the Asia–Pacific region. The Asia–Pacific Metabolic and Bariatric Surgery Society (APMBSS) 2010 Survey reported that among 2091 surgical cases in 11 Asian countries (excluding Australia) in 2009, AGB accounted for 35.6% of the procedures, RYGB accounted for 27.7%, and SG accounted for 24.8% [19]. However, the latest IFSO survey demonstrated that SG accounted for 54% of the procedures, OAGB accounted for 20%, and RYGB accounted for 13% in the 11 IFSO-APC countries [8].

Due to the COVID-19 pandemic, the IFSO announced a position statement, “Recommendations for metabolic and bariatric surgery during the COVID-19 pandemic from IFSO,” in May 2020 [20]. The general recommendations were that all elective surgical and endoscopic cases for metabolic and bariatric surgery should be postponed during the pandemic. In addition, clinic and hospital visits were not recommended for follow-up. Based on the results from a worldwide survey, the COVID-19 pandemic had a strong impact on bariatric practice with respect to surgical and outpatient planning, as well as personnel management [21]. The Australian & New Zealand Metabolic and Obesity Surgery Society repeatedly announced the guidelines for bariatric surgery during the COVID-19 pandemic on their website [22]. Singapore bariatric surgeons reported their methods for the management of pre- and post-bariatric patients during the COVID-19 pandemic [23]. The Chinese Society for Metabolic and Bariatric Surgery also reported their methods for the management of bariatric surgery patients during the COVID-19 pandemic [24]. Their reports included a detailed schematic illustration of algorithms for both clinic and admission processes. Then, the Obesity and Metabolic Surgery Society of India published their recommendations for bariatric and metabolic surgery practice during the COVID-19 pandemic [25]. They reported the requirements and precautions for restarting bariatric and metabolic surgery, with an emphasis on safe delivery and high-quality care. In this way, the IFSO-APC countries and regions dealt with the COVID-19 pandemic. Ultimately, annual surgical cases decreased by 24.9% in 2020 in comparison to 2019. This was obviously due to the reduction of operations in India, Malaysia, Singapore, and Philippines, in which the government executed a policy of temporary restriction of access to bariatric/metabolic surgery during the COVID-19 pandemic.

In 2010, bariatric and metabolic surgeries were not covered by public health insurance in 9 of the 13 IFSO-APC countries and regions (69.2%). However, in 2020, Japan, Korea, Hong Kong, Thailand, and India—which were among the 9 countries, provided public health insurance coverage for both bariatric and metabolic surgeries. In Korea, Song et al. showed the cost-effectiveness of bariatric surgery through a retrospective study in 2013 [26]. A prospective multicenter clinical trial was conducted among 13 Korean university hospitals in conjunction with the Korean Society for Metabolic and Bariatric Surgery. It showed that bariatric surgery in patients with BMI ≥ 30 kg/m^2^ was cost-effective and more effective than non-surgical treatment for the reduction of BMI and remission of obesity-related comorbidities [27, 28]. Based on the results, public health care insurance covered bariatric/metabolic surgery for patients with BMI ≥ 30 kg/m^2^ in January 2019. In Thailand, the Thai Society for Metabolic and Bariatric Surgery announced the consensus guidelines on bariatric surgery in 2018 [29], and also proposed government insurance coverage of bariatric/metabolic surgery. The insurance coverage was decided to be approved in 2020 and has been approved since April 2021. The Japanese Society for Treatment of Obesity has repeatedly engaged in public insurance coverage of bariatric/metabolic surgery. In patients with BMI ≥ 35 kg/m^2^, laparoscopic SG has been fully covered since April 2014, and SG with duodenojejunal bypass has been partially covered since March 2018. Recently, a report from Hong Kong showed that bariatric surgery in patients with BMI ≥ 27.5 kg/m^2^ and T2DM improved obesity-related comorbidities over the long term but was not cost-effective [30].

Current issues in relation to bariatric/metabolic surgery in the IFSO-APC countries and regions include public insurance coverage and medical funding systems, a lack of awareness and comprehension among physicians and the general public, and the small number of surgical cases. In particular, a lack of awareness and comprehension regarding bariatric/metabolic surgery among physicians and the general public remains problematic in Asian countries. In this survey, 3 of the 13 countries (23.1%) cited raising awareness about bariatric/metabolic surgery as the biggest change in the 10-year period, but 5 countries (38.5%) pointed out a lack of awareness and comprehension as a current problem. The previous APMBSS survey also pointed out this problem [31], and the situation seems to be related to culture and religion. In addition, unfortunately, there is also an obesity stigma among the public [32]. Many pieces of evidence supporting the safety and effectiveness of bariatric/metabolic surgery have been published around the world [33]. However, physicians and the general public in Asian countries are still not sufficiently aware of the safety and effectiveness of bariatric/metabolic surgery. Currently, studies from European countries and the USA have reported that primary care physicians remain reluctant to refer their patients for bariatric/metabolic surgery. This may be associated with a lack of knowledge [34–37]. In the results of an interview with the general public in Saudi Arabia, most participants recognized the association between obesity and its comorbidities. However, more than 20% of the participants were unaware of bariatric/metabolic surgery [38]. In addition, approximately 20% of participants considered it to be a cosmetic procedure, and approximately 50% were unaware of the indications for bariatric/metabolic surgery. Fan et al. reported on Chinese nurses’ knowledge and attitudes towards obesity and bariatric surgery [39]. The nurses generally had high knowledge in relation to obesity and T2DM, but only less than 50% of the nurses knew about the relationship between obesity and other related diseases, such as carcinoma and gastroesophageal reflux disease. In addition, their acceptance of the safety and efficacy of bariatric surgery was shallow (< 30%). They suggested that it is crucial to enhance the continuous education of Chinese nurses in relation to obesity and bariatric surgery. A certified bariatric nurse program has already been established in the American Society for Metabolic and Bariatric Surgery [40]. This survey indicated that the Taiwan Society for Metabolic and Bariatric Surgery recently started a similar program. Such programs may be instrumental in educating nurses.

The present study was associated with some limitations. The collection of data was somewhat incomplete because a considerable number of countries did not have nationwide registries by 2010, and 2020 was in the middle of the COVID-19 pandemic. Nonetheless, this study demonstrated that bariatric/metabolic surgery has rapidly developed concomitant with the changes in supportive social systems in Asia–Pacific countries and regions over the last 10 years. The IFSO-APC is dedicated to advancing bariatric/metabolic surgery in the Asia–Pacific region.

In conclusion, bariatric/metabolic surgery has rapidly grown in the Asia–Pacific region for the last 10 years, primarily due to the expansion of surgical criteria and establishment of public health insurance system based on the dedication of the IFSO and IFSO-APC members in each country and region.

## References

[1] The IFSO-APC&JSSO Congress 2011 program. Japanese Society for Treatment of Obesity homepage. http://plaza.umin.ne.jp/~jsto/information/ifso_apc/index.html

[2] Kasama K, Mui W, Lee WJ (2012). IFSO-APC consensus statements 2011. Obes Surg.

[3] Scopinaro N. The IFSO and obesity surgery throughout the world. International Federation for the Surgery of Obesity. Obes Surg 1998;8(1):3–8 10.1381/096089298765554971. 10.1381/096089298765554971 9562479

[4] Buchwald H, Williams SE (2004). Bariatric surgery worldwide 2003. Obes Surg.

[5] Buchwald H, Oien DM (2009). Metabolic/bariatric surgery worldwide 2008. Obes Surg.

[6] Buchwald H, Oien DM (2013). Metabolic/bariatric surgery worldwide 2011. Obes Surg.

[7] Angrisani L, Santonicola A, Iovino P, Formisano G, Buchwald H, Scopinaro N (2015). Bariatric surgery worldwide 2013. Obes Surg.

[8] Angrisani L, Santonicola A, Iovino P, Ramos A, Shikora S, Kow L (2021). Bariatric surgery survey 2018: similarities and disparities among the 5 IFSO chapters. Obes Surg.

[9] Prevalence of obesity among adults, BMI ≥ 30, age-standardized estimates by country, Global Health Observatory (GHO) data. World Health Organization homepage. http://apps.who.int/gho/data/node.main.A900A?lang=en

[10] Chang HC, Yang HC, Chang HY (2017). Morbid obesity in Taiwan: prevalence, tends, associated social demographics, and lifestyle factors. PLoS One.

[11] Ko GTC, Tang JSF (2006). Prevalence of obesity, overweight and underweight in a Hong Kong community: the United Christian Nethersole Community Health Service (UCNCHS) primary health care program 1996–1997. Asia Pac J Clin Nutr.

[12] Body Mass Index (BMI) Distribution, Centre for Health Protection, Department of Health, the Government of the Hong Kong Special Administrative Region homepage. https://www.chp.gov.hk/en/statistics/data/10/280/6620.html

[13] Appendix 1, Country summary table: estimates for 2010. IDF Diabetes Atlas 4th edition, 82–95, IDF homepage. http://diabetesatlas.org/atlas/fourth-edition/

[14] Appendices, country summary tables. IDF Diabetes Atlas 2021, 104–127, IDF homepage. http://diabetesatlas.org/atlas/tenth-edition/

[15] Du X, Dai R, Su MI (2016). Bariatric surgery in China: how is this new concept going?. Obes Surg.

[16] Kosai N, Rajan R (2018). History and progress of bariatric surgery in Malaysia. Obes Surg.

[17] Ohta M, Kasama K, Sasaki A (2021). Current status of laparoscopic bariatric/metabolic surgery in Japan: the sixth nationwide survey by the Japan Consortium of Obesity and Metabolic Surgery. Asian J Endosc Surg.

[18] Bulugahapitiya U, Wijeratne T, Jayawardena R (2021). The first Sri Lankan experience on laparoscopic bariatric surgery. Obes Surg.

[19] Lomanto D, Lee WJ, Goel R (2012). Bariatric surgery in Asia in the last 5 years (2005–2009). Obes Surg.

[20] Yang W, Wang C, Shikora S, Kow L (2020). Recommendations for metabolic and bariatric surgery during the COVID-19 pandemic from IFSO. Obes Surg.

[21] Lazaridis II, Kraljević M, Schneider R (2020). The impact of the COVID-19 pandemic on bariatric surgery: results from a worldwide survey. Obes Surg.

[22] COVID-19 Guidelines. The ANZMOSS site: https://anzmoss.com.au/covid-19-guidelines/

[23] Yeo C, Ahmed S, Oo AM, Koura A, Kaushal Sanghvi K, Yeo D (2020). COVID-19 and obesity-the management of pre- and post-bariatric patients amidst the COVID-19 pandemic. Obes Surg.

[24] Dong Z, Zhang P, Zhu J, Bai J (2020). Recommendations to manage patients for bariatric surgery in the COVID-19 pandemic: experience from China. Obes Surg.

[25] Aggarwal S, Mahawar K, Khaitan M (2020). Obesity and Metabolic Surgery Society of India (OSSI) recommendations for bariatric and metabolic surgery practice during the COVID-19 pandemic. Obes Surg.

[26] Song HJ, Kwon JW, Kim YJ, Oh SH, Heo Y, Han SM (2013). Bariatric surgery for the treatment of severe obese patients in South Korea – is it cost effective?. Obes Surg.

[27] Comparative study in morbidity obese patient with surgical and medical treatments: effectiveness, safety and cost-effectiveness R&D Report. Korean Ministry of Health and Welfare Affairs. Korea Health Industry Development Institute.; Available from: https://www.khidi.or.kr/kps/researchInfo/list?menuId=MENU02230&searchContinuStYear=&searchSprcRsrhInttNm=&searchFlnmKrn=&searchGwrd=&searchPjtMngmNo= HC15C1322&searchPjtNm=#.

[28] An S, Park HY, Oh SH (2020). Cost-effective of bariatric surgery for people with morbid obesity in South Korea. Obes Surg.

[29] Techagumpuch A, Pantanakul S, Chansaenroj P (2020). Thai Society for Metabolic and Bariatric Surgery Consensus Guideline on bariatric surgery for the treatment obese patient in Thailand. J Med Assoc Thai.

[30] Wu T, Wong SKH, Law BTT (2021). Bariatric surgery is expensive but improves co-morbidity: 5-year assessment of patients with obesity and type 2 diabetes. Br J Surg.

[31] Ohta M, Seki Y, Wong SK (2019). Bariatric/metabolic surgery in the Asia-Pacific region: APMBSS 2018 Survey. Obes Surg.

[32] Rubino F, Puhl RM, Cummings DE (2020). Joint international consensus statement for ending stigma of obesity. Nat Med.

[33] Rubino F, Nathan DM, Eckel RH (2016). Metabolic surgery in the treatment algorithm for type 2 diabetes: a joint statement by International Diabetes Organizations. Diabetes Care.

[34] Major P, Stefura T, Jezierska-Kazberuk M (2016). The knowledge of Polish primary care physicians about bariatric surgery. Wideochir Inne Tech Maloinwazyjne.

[35] Conaty EA, Denham W, Haggerty S, Linn JG, Joehl JG, Ujiki MB (2020). Primary care physicians’ perceptions of bariatric surgery and major barriers to referral. Obes Surg.

[36] Egerer M, Kuth N, Koch A (2021). General practitioner's knowledge about bariatric surgery is associated with referral practice to bariatric surgery centers. Int J Environ Res Public Health.

[37] Memarian E, Carrasco D, Thulesius H, Calling S (2021). Primary care physicians’ knowledge, attitudes and concerns about bariatric surgery and the association with referral patterns: a Swedish survey study. BMC Endocr Disord.

[38] Altaf A, Abbas MM (2019). Public perception of bariatric surgery. Saudi Med J.

[39] Fan M, Cheung PN, Tang S (2020). Knowledge and attitudes towards obesity and bariatric surgery in Chinese nurses. Obes Surg.

[40] Certified Bariatric Nurse (CBN). The ASMBS site: https://asmbs.org/professional-education/cbn

